# THETA system allows one-step isolation of tagged proteins through temperature-dependent protein–peptide interaction

**DOI:** 10.1038/s42003-019-0457-8

**Published:** 2019-06-14

**Authors:** Kota Miura, Yusuke Tsuji, Hiromasa Mitsui, Takuya Oshima, Yosei Noshi, Yudai Arisawa, Keiko Okano, Toshiyuki Okano

**Affiliations:** Department of Electrical Engineering and Bioscience, Graduate School of Sciences and Engineering, Waseda University, TWIns, Wakamatsucho 2-2, Shinjuku-Ku, Tokyo 162-8480 Japan

**Keywords:** Biomaterials - proteins, Antibody isolation and purification

## Abstract

Tools to control protein-protein interactions by external stimuli have been extensively developed. For this purpose, thermal stimulation can be utilized in addition to light. In this study, we identify a monoclonal antibody termed C13 mAb, which shows an approximately 480-fold decrease in the affinity constant at 37 °C compared to that at 4 °C. Next, we apply this temperature-dependent protein-peptide interaction for one-step protein purifications. We term this THermal-Elution-based TAg system as the THETA system, in which gel-immobilized C13 mAb-derived single-chain variable fragment (scFv) (termed THETAL) is able to bind with proteins tagged by C13 mAb-epitope(s) (THETAS) at 4 °C and thermally release at 37–42 °C. Moreover, to reveal the temperature-dependent interaction mechanism, molecular dynamics simulations are performed along with epitope mapping experiments. Overall, the high specificity and reversibility of the temperature-dependent features of the THETA system will support a wide variety of future applications such as thermogenetics.

## Abstract (web summary)

Kota Miura et al. develop the THermal-Elution-based TAg (THETA) system that allows one-step isolation of tagged proteins. The temperature-dependent protein-peptide interaction used in the THETA system ensures high specificity for protein purification.

## Introduction

Artificial manipulation of biological molecules constitutes an extremely powerful tool in life science research. For such purposes, light or thermal stimulus has generally been used to activate caged compounds with photoreactive groups or thermo-sensitive molecules. Optogenetics, as exemplified by the photic control of neuronal activities by local light irradiation to genetically introduced light-sensitive ion channels in living animals, has led a new avenue in brain science^1^. However, in contrast to the number of photosensitive proteins being utilized in optogenetics, only a few genetic resources are being used in thermogenetics (e.g., heat shock protein genes and temperature-dependent TRP channels)^2,3^. This may partly occur because proteins are more problematic than DNAs in terms of thermal manipulation. Nucleotides intrinsically retain their temperature-dependent characteristics upon interconversion between single- and double-stranded nucleotides, and this property allows novel technologies such as DNA computing^4^ in addition to common methodologies such as nucleic acid probing. Therefore, a novel protein element for the thermal control of protein–protein interaction without requiring denaturation within a physiological temperature range would allow a wide variety of biochemical and biomedical applications.

It is widely recognized that a fraction of antibodies show temperature dependencies in their binding to the antigen^5,6^. Such temperature dependency is, however, scarcely utilized for the thermal control of protein interactions, likely because the change in their affinity is too small for such purpose. Rather, monoclonal antibodies (mAbs) showing temperature dependency are routinely specifically excluded in the course of screening owing to their inconvenience in use^7^. Alternatively, in the present study, we isolate an mAb exhibiting extraordinary large temperature dependency in the immunoreaction and develop the THermal-Elution-based TAg (THETA) system, a practical system in which protein–peptide interaction is controlled thermally. This system enables immunoaffinity purification of tagged recombinant proteins by elution from an immunoglobulin- or its single-chain variable fragment (scFv)-immobilized column with only temperature change in a physiological range. The molecular mechanism of the temperature-dependent interaction is further combinatorially analyzed by epitope analysis and molecular dynamics (MD) simulations.

## Results

### Antibody with temperature-dependent affinity

**Fig. 1:**
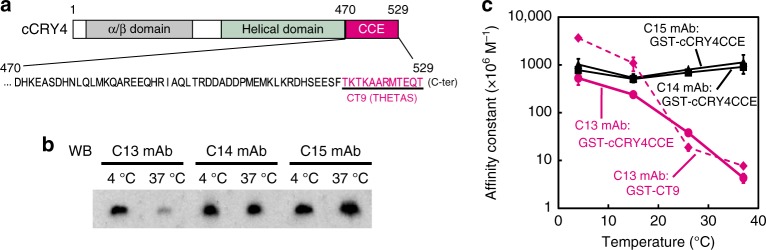
Temperature dependency of the C13 monoclonal antibody. **a** cCRY4 C-terminal extension (CCE) sequence and epitope locations for the C13 monoclonal antibody. **b** Western blot analysis of GST-cCRY4CCE using C13–C15 anti-cCRY4CCE antibodies. The first antibody reactions were performed at 4 °C or 37 °C and the 2nd were performed at 4 °C. Overall picture is shown in Supplementary Fig. 2a. **c** Temperature dependencies in affinity constants for C13–C15 mAbs to GST-cCRY4CCE or GST-CT9. The affinity constants were measured by indirect competitive ELISA at 4, 15, 26, and 37 °C. Error bars represent standard deviation (*n* = 4)

In the course of biochemical and physiological analysis of a blue light photoreceptor chicken cryptochrome 4 (cCRY4), we developed 15 mAbs termed C1–C15 against the C-terminal region (cCRY4CCE) (Fig. 1a and Supplementary Fig. 1a). Notably, we found that among these antibodies, the detection sensitivity of C13 mAb in western blot markedly decreased at 37 °C compared to that at 4 °C (Fig. 1b and Supplementary Fig. 2a). We confirmed that this decrease is not caused by degradation or irreversible instability of the C13 mAb at 37 °C (Supplementary Fig. 3), then measured affinity constants at 4–37 °C by indirect competitive ELISA (Fig. 1c and Supplementary Table 1). Although the affinity constants of C14 and C15 mAbs were almost constant in this temperature range, C13 mAb demonstrated about 120-fold higher affinity at 4 °C than that at 37 °C. Competitive ELISA (Supplementary Fig. 1b) using nine synthetic peptides (CT1–CT9; Supplementary Fig. 1a) localized the epitope of C13 mAb within the last C-terminal 12 amino acids of cCRY4 (TKTKAARMTEQT; CT9 in Supplementary Fig. 1a and hereafter termed THETAS). Notably, the affinity constant for C13 mAb against GST–CT9, a fusion protein of glutathione *S*-transferase (GST) and CT9, was ~480 times higher at 4 °C than that at 37 °C; this value likely indicated C13 mAb as exhibiting the strongest temperature dependency among those ever reported for the other mAbs. A carboxyl terminally extended peptide (CT9-Y, TKTKAARMTEQTY), albeit slightly weaker than Y-CT9, provided effective competition (Supplementary Fig. 1c), indicating that the terminal carboxyl group is not essential for the immunoreaction by C13 mAb.

### Affinity purification of cCRY4 by thermal elution

**Fig. 2:**
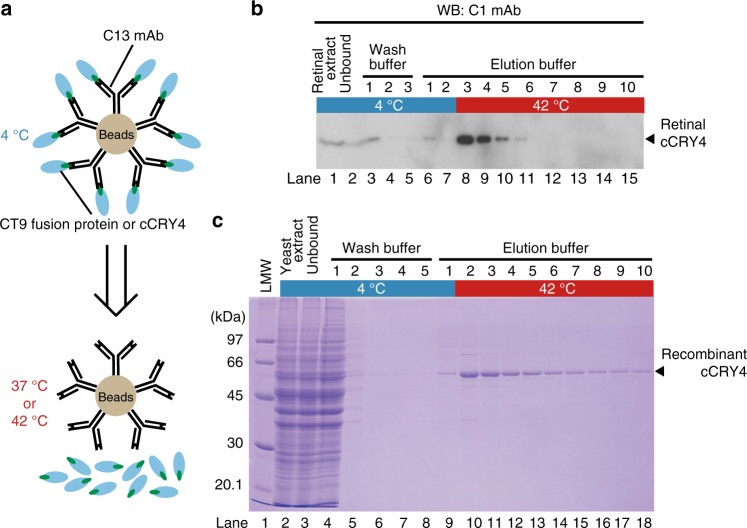
Affinity purification of cCRY4 by thermal elution from C13-mAb-gel. **a** Schematic drawing for the thermal elution of cCRY4 or CT9-fused proteins from C13-mAb-gel. **b** Affinity purification of retinal cCRY4 from chick retinas by thermal elution. Soluble fraction extracted from 50 retinas was applied to 1 mL of C13-mAb-gel, and the gel was washed three times with 5 mL of wash buffer at 4 °C, followed by successive treatments with elution buffer twice at 4 °C and eight times at 42 °C. The column fractions were analyzed by western blotting using C1 mAb as the primary antibody. Overall picture is shown in Supplementary Fig. 2b. **c** Affinity purification of recombinant cCRY4 expressed in yeast by thermal elution. The recombinant cCRY4 was purified from 5 L of yeast culture similar to that described in **b**, and the fractions were analyzed by SDS-PAGE/Coomasie brilliant blue staining. The calculated molecular mass of cCRY4 is 61,063 Da

Taking advantage of the temperature dependency of the C13 mAb, we next examined whether this mAb could be utilized to trap the antigenic polypeptides at low temperature and release them by raising the temperature (Fig. 2a). Because cCRY4 is relatively abundant in soluble extracts of the chick retina^8^ and the budding yeast MaV203 overexpressing cCRY4^9^, these extracts were used as starting materials (Fig. 2b, c, and Supplementary Fig. 2b). After loading either of the extracts and washing at 4 °C, native or non-tagged cCRY4 was eluted with elution buffer at 42 °C (lanes 8–11 in Fig. 2b, lanes 10–18 in Fig. 2c). CBB staining of the fractions (Fig. 2c) suggested that the non-tagged cCRY4 could be highly purified from the yeast extract solely by one-step immunoaffinity column chromatography.

**Fig. 3:**
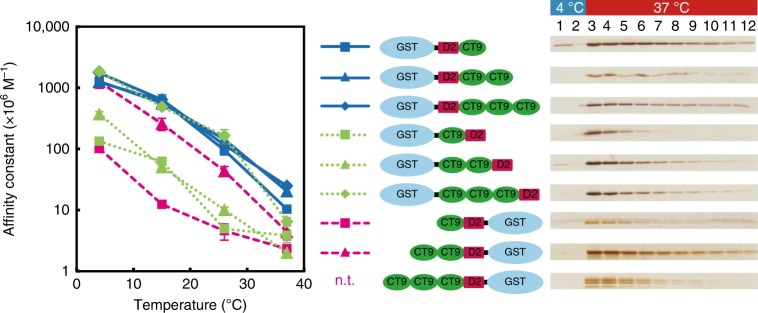
Thermal-elution-based purification and affinity constants of C13 mAb and CT9 fusion proteins. Various CT9 fusion proteins were purified from *E. coli* extract, separated by SDS-PAGE, and subjected to silver staining (overall pictures are shown in Supplementary Fig. 4). The affinity constants between the C13 mAb and each CT9 fusion protein were measured by indirect competitive ELISA at 4, 15, 26, and 37 °C. CT9 × 3-D2-GST was not used for analysis of affinity constants because of contamination with many degraded products (CT9x1-D2-GST and CT9x2-D2-GST). Error bars represent standard deviation (*n* = 3)

We further examined whether CT9 is suitable for general utility as a tag for protein purification. As the carboxyl group at the C-terminal Thr of CT9 is not essential for its binding to C13 mAb (Supplementary Fig. 1c), CT9 was expected to be immunoreactive even when fused at the N terminus or tandemly repeated. Therefore, we prepared nine recombinant GST proteins tagged with 1–3 repeats of CT9 at or near the C terminus or at the N terminus (Fig. 3 and Supplementary Fig. 4). Soluble protein extract of *Escherichia coli* expressing each fusion protein was loaded on a C13-mAb-immobilized gel, from which the fusion protein was selectively eluted at 37 °C regardless of the position of CT9 and the number of repeats (×1, ×2, and ×3). Measurement of affinity constants of the C13 mAb to each purified fusion protein (Fig. 3) showed that all the examined antigens retain temperature dependency (Fig. 3, and Supplementary Table 2). No large change in the affinity constants was observed when the number of repeats was increased up to three at the C terminus (Fig. 3, blue lines). Conversely, when a decapeptide (D2; EFSHRGSQRN) was fused at the C terminus, the affinity constants decreased to about 1/10 (Fig. 3, green dotted line with boxes), whereas they recovered to levels of the C-terminal fusions (Fig. 3, blue lines) by prior repetition of three CT9 copies (Fig. 3, green dotted line with diamonds). Such positional attenuation was also observed when D2 was fused at the N terminus of CT9 (Fig. 3, red dashed line with squares), which was recovered by two CT9 repetitions (Fig. 3, red dashed line with triangles).

### Expression, purification, and renaturation of C13scFv and its application to thermal-elution-based affinity purification

**Fig. 4:**
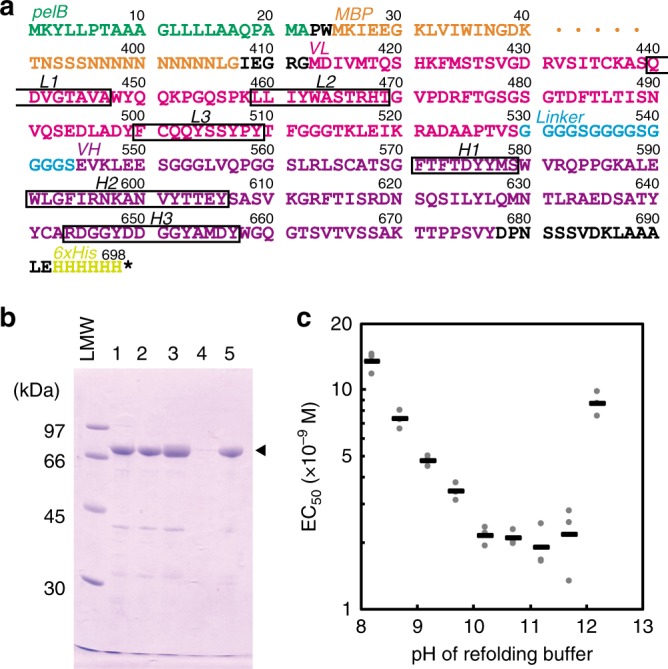
Construction, purification, and renaturation of MBP-C13scFv-His. **a** Amino acid sequences of MBP-C13scFv-His. The region of 413–677 corresponds to THETAL (see below). **b** Purification of MBP-C13scFv-His. Lane 1, insoluble fraction obtained after sonication of *E. coli* BL21(DE3) overexpressing MBP-C13scFv-His; lane 2, insoluble fraction after washing with detergents; lane 3, urea-extracted fraction; lane 4, residual pellet after extraction by urea; lane 5, refolded MBP-C13scFv-His. **c** Binding activities of MBP-C13scFv-His refolded at various pH. MBP-C13scFv-His was refolded by using refolding buffer adjusted to different pH. After dialysis against PBS (pH 7.4), the binding activities to cCRY4CCE were measured by ELISA using anti-6xHis antibody (Wako), changing the concentration of MBP-C13scFv-His. The half-maximal effective concentrations (EC_50_) of the immunoreactions were calculated by fitting the value to the Rodbard model using ImageJ. The numbers of observations are three

scFv is a small protein composed of the variable regions of an antibody and a short linker peptide. Considering the wide availability of scFvs, we isolated cDNA encoding variable regions of C13 mAb from the hybridoma and constructed an *E. coli* expression vector for the scFv of C13 mAb (C13scFv, termed “THETAL” hereafter) fused with a maltose-binding protein (MBP) and 6 × His-tag at the N terminus and C terminus, respectively (MBP-C13scFv-His, Fig. 4a). A large quantity of the MBP-C13scFv-His protein was found in the insoluble fraction after sonication of MBP-C13scFv-His-expressing *E. coli* cells (Fig. 4b, lane 1) and the detergent-treated insoluble fraction (Fig. 4b, lane 2). Most contaminating proteins were removed by the above procedures; thus, MBP-C13scFv-His had been purified to near homogeneity and was then solubilized using 8 M urea (Fig. 4b, lanes 3, 4). In the course of optimizing the conditions for refolding MBP-C13scFv-His, we found that the pH of the refolding buffer comprised an important determinant for the efficiency of functional refolding of MBP-C13scFv-His, which was high in the basic pH range (pH 10.2–11.7) (Fig. 4c), a rather basic value compared to those commonly used for refolding of scFv (pH 8.0–8.5)^10–14^. We obtained ~200 mg of MBP-C13scFv-His with purity close to 90% (Fig. 4b, lane 5) per liter of *E. coli* culture without using column chromatography.

**Fig. 5:**
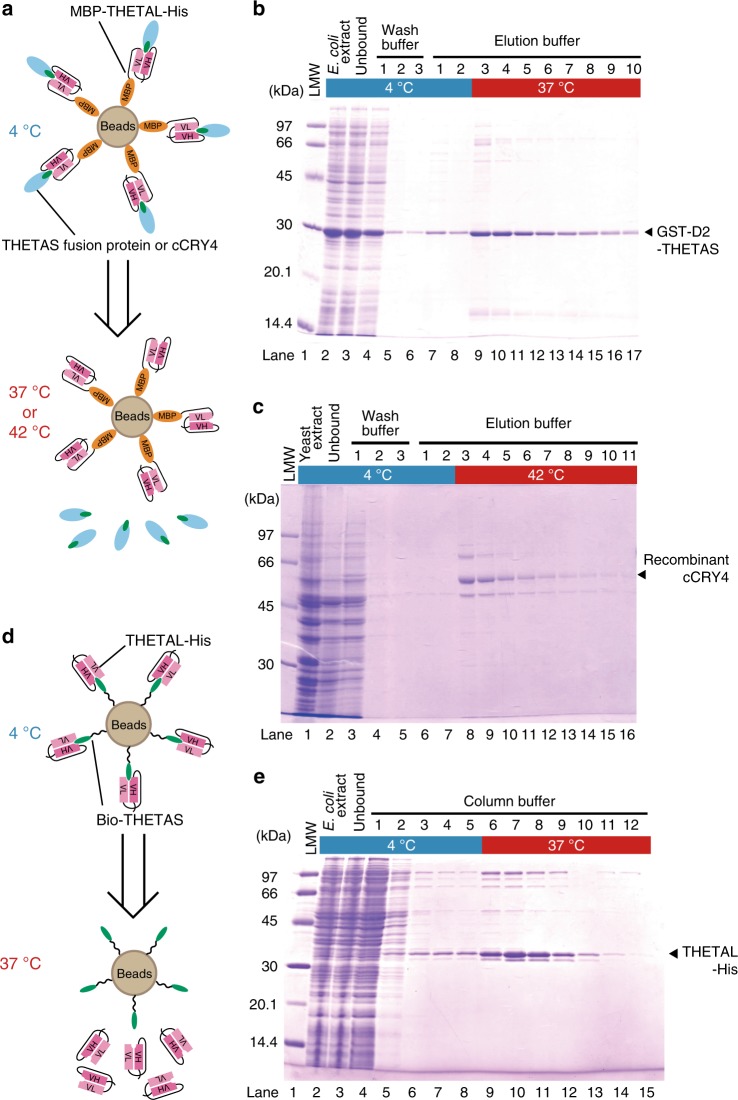
Thermal-elution-based purification of THETAS-containing proteins and THETAL by the THETA system. **a** Schematic drawing for thermal-elution-based purification of THETAS-tagged protein or cCRY4 using MBP-THETAL-His-gel. **b** GST-D2-CT9 (THETAS) was thermo-purified from an *E. coli* extract from 500 mL of culture using MBP-THETAL-His-gel. **c** cCRY4 was purified from 5 L culture of budding yeast using MBP-THETAL-His-gel. **d** Schematic drawing for thermal-elution-based purification of THETAL-His using THETAS-gel. **e** THETAL-His was purified by the THETA system from 500 mL of *E. coli* culture using THETAS-gel

Next, we examined whether the MBP-C13scFv-His-immobilized gel was applicable to thermal-elution-based affinity purification (Fig. 5a). MBP-C13scFv-His efficiently trapped GST-D2-CT9 similarly to C13 mAb-immobilized gel and released the peptide upon warming the column (Fig. 5b), suggesting that C13 mAb could be replaced with C13scFv. Therefore, we designated this THETA system as being composed of a THETA-large component (THETAL; i.e., C13scFv) and a THETA-small component (THETAS; i.e., CT9 epitope-tag).

cCRY4 constitutes a leading candidate molecule for a magnetoreceptor that plays a pivotal role in a light-dependent chemical compass in birds^8,15–17^. Accordingly, a method for obtaining large amounts of CRY4 proteins for elucidation of the molecular mechanism of magnetoreception is highly anticipated. To address the suitability of the THETA system for this purpose, a full-length untagged cCRY4, which intrinsically contains THETAS, was expressed in budding yeast and purified by the THETA system with a yield of ~100 μg per liter of culture (Fig. 5c). Ultraviolet–visible spectroscopic analysis (Supplementary Fig. 5) revealed binding of the FAD chromophore and cCRY4 photoreactivity as we had reported previously^9^.

To extend applicability of THETA system, we further examined whether THETAL and THETAS were interchangeable by using a THETAS-immobilized gel to isolate THETAL (Fig. 5d). Biotinylated THETAS (bio-THETAS, Fig. 5d) was immobilized on streptavidin sepharose (GE Healthcare), to which the extract of *E. coli* expressing THETAL-His was added at 4 °C, and then the bound THETAL-His was recovered by raising the temperature (Fig. 5e).

### Analysis of temperature-dependent antigen–antibody interaction mechanism by combining mutant analysis and MD simulation

**Fig. 6:**
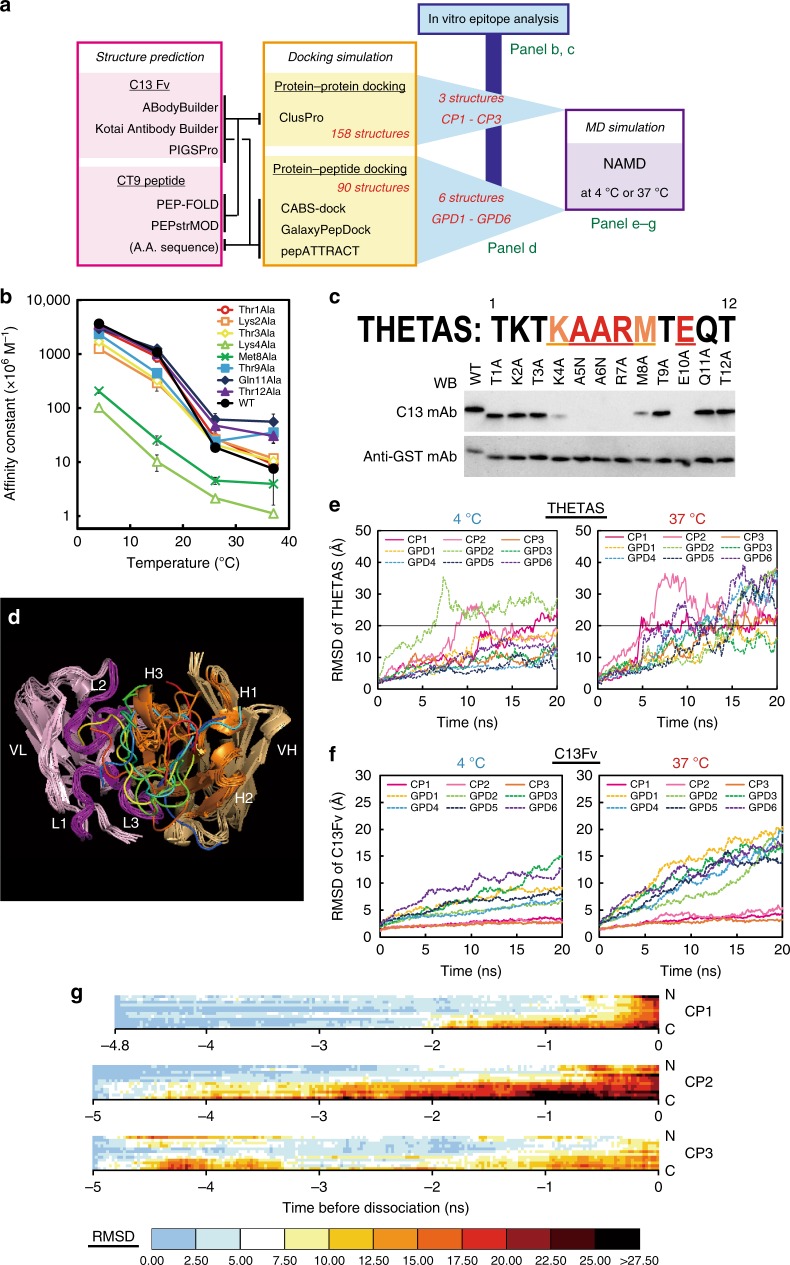
Analysis of the temperature-dependent interaction between C13Fv and THETAS. **a** Flowchart depicting the analysis. Predicted structures of C13Fv and THETAS were used for docking simulation together with amino acid information of THETAS. Among the resultant 248 docked structures, nine structures (**d**) were selected in which five or six important amino acids (**b**, **c**) are involved in the binding. **b** Affinity constants between C13 mAb and THETAS mutants measured by indirect competitive ELISA at 4, 15, 26, and 37 °C. Error bars represent standard deviation (*n* = 3). **c** Epitope analysis by western blot of mutated THETAS. Overall pictures are shown in Supplementary Fig. 2c, d. **d** Superimposed drawing of nine selected docking structures. H chain, L chain, CDRs of H chain, and CDRs of L chain are colored in light orange, light pink, orange, and purple, respectively. Nine predicted structures of THETAS are differently colored. **e**, **f** Temporal changes in averaged RMSDs of THETAS (**e**) or C13Fv (**f**) in the MD simulation. **g** Heatmap showing the RMSDs from 5 ns prior to THETAS dissociation in the MD simulation. Dissociation of THETAS from C13Fv was judged when the average value of RMSD of α carbon for each amino acid of THETAS exceeded 20 Å by a simple expedient

To explore the molecular mechanism underlying the core technology of the THETA system; i.e., the temperature-dependent interaction between THETAL and THETAS, we performed in silico docking and MD simulations with the aid of in vitro epitope mapping using mutated THETAS (Fig. 6a). First, structures of the C13 mAb variable region (C13Fv as compatible substitutes of THETAL) and THETAS were inferred by three and two kinds of prediction servers, respectively (Fig. 6a)^18–22^. We obtained 248 C13Fv:THETAS docking structures by combinatorial analyses of predicted structures of C13Fv and those of THETAS (or its sequence information) by using four kinds of docking simulation tools (Fig. 6a)^23–27^. In parallel, we prepared 12 kinds of GST-THETAS-fusion proteins in which each of the 12 amino acids within THETAS was substituted by alanine (or asparagine when the amino acid residue was alanine) to evaluate the importance in the interaction with C13 mAb (Fig. 6b, c and Supplementary Table 3). In western blot analysis using C13 mAb (Fig. 6c, Supplementary Fig. 2c,  d), the immunoreactivity was completely lost in the Ala5Asn, Ala6Asn, Arg7Ala, and Glu10Ala mutants and weakened in Lys4Ala and Met8Ala mutants (where numbers reflect the position in THETAS). These results indicated that these six amino acid residues are indispensable for or substantively contribute to the binding to THETAL and hence likely form the core of the C13 epitope. This speculation was confirmed by competitive ELISA, from which it was additionally revealed that the affinity constants of the Thr9Ala, Gln11Ala, and Thr12Ala mutants are higher than that of wild-type at 37 °C (Fig. 6b).

Considering the likely core C13 epitope, we selected the structures in which five or six of these six amino acids are involved in the interaction from among the 248 predicted C13Fv:THETAS structures. Three and six structures generated by ClusPro (CP1–CP3) and GalaxyPepDock (GPD1–GPD6) satisfied this constraint, respectively (Supplementary Table 4). Notably, in all nine selected C13Fv:THETAS structures, THETAS adopted disordered random coil configurations and docked mainly with the H3 loop within the complementarity determining region, although the predicted binding sites of THETAS were not highly conserved among the nine structures (Fig. 6d and Supplementary Fig. 6).

Next, in order to interrogate a possible temperature dependency in the structural stability of the predicted THETAL:THETAS complexes, we conducted MD simulations at 4 or 37 °C starting from each of the nine C13Fv:THETAS complex structures (the results of CP1–CP3 are described on Supplementary Movies 1–3). The structural stability of C13Fv and dissociation of THETAS from C13Fv was evaluated by root mean square deviation (RMSD) of C13Fv and THETAS (Fig. 6e, f, Supplementary Figs. 7 and 8). Owing to the difficulty in precisely judging the dissociation, we defined an expedient criterion whereby THETAS was considered to be dissociated from C13Fv when the RMSD of THETAS exceeded 20 Å. We found that THETAS was dissociated from C13Fv within 20 ns at 37 °C in all nine predictions. THETAS dissociated slower at 4 °C than 37 °C in two predictions and never dissociated within 20 ns in six predictions, although THETAS dissociated faster at 4 °C than 37 °C in one case (Supplementary Table 4). Moreover, the structure of C13Fv apparently collapsed at 37 °C along with the dissociation of THETAS in the models of GPD1–GPD6; consistent with this, RMSD of C13Fv became much larger in those models than in the models of CP1–CP3 (Fig. 6f). Because the thermal interaction in the THETA system is reversible, we considered that the models of CP1–CP3 are preferable for use in the speculation of mechanisms for thermal dissociation of THETAS from C13Fv. Notably, RMSD of α carbon for each of the 12 amino acids in THETAS at 37 °C from 5 ns prior to the dissociation (Fig. 6g) showed that the dissociation likely initiates by detachment near the C terminus of THETAS and completes within a few ns (Fig. 6g).

## Discussion

In this study, we established the THETA system, a practical protein/peptide-tag system applicable to the thermal control and purification of any protein of interest. The THETA system offers many advantages compared with conventional affinity tag systems: high specificity based on the antigen–antibody reaction, a small peptide tag as short as Strep-tag and His-tag, affinity varying over 100-fold in a range of physiological temperature, milder elution conditions than pH change, no addition of peptide, ligand, or salt in the elution buffer, enabling direct reloading of the eluate from the THETAL-immobilized column to the same column. By genetic modification or gene transfer, the THETAL:THETAS interaction may also be utilized in in vivo applications such as thermal drug delivery, thermogenetics, and thermal control of cellular dynamics. In this regard, THETAL has the relative merits of an scFv compared with the parent IgG. In our present protocol, elution of THETAS-tagged proteins is completed within 30–60 min (incubation for 10–30 min and the elution for 20–30 min). An ELISA showed that the MBP-THETAL-His activity is kept at ~86% after 30 min incubation at 37 °C, and therefore this is not practically problematic considering the large binding capacity of the resin. In fact, we routinely reuse the same MBP-THETAL-His-immobilized gel to purify cCRY4 or THETAS-fusion proteins.

As an example of a protein purification method utilizing temperature change, elastin-like polypeptide tag, a repeat sequence motif of Val-Pro-Gly-Xaa [not Pro]-Gly that forms an aggregate depending on temperature, has been reported^28–30^. Notably, because THETAS is expected structurally to be more soluble than elastin-like polypeptide, THETAS may be more suitable for future application to in vivo thermogenetics.

It is also important to compared purification efficiency of this system with that of other tag systems such as His-tag. As an initial assessment, we expressed a model protein GST-EYFP-His_6_-THETAS in *E. coli* and compared the purification efficiency (purities of the eluates and recoveries) between THETA and Ni-NTA systems (Supplementary Fig. 9). The purities of main eluates were higher for THETA system (46–49%) than for Ni-NTA system (34–37%), although the recoveries of the loaded protein were 11.4% and 41% for the THETA and Ni-NTA systems, respectively. Therefore, we consider the THETA system will be of practical importance as well as the other conventional affinity purification methods.

The temperature dependency of C13mAb (120–480 times, Fig. 1c) is extraordinarily large and therefore suitable for the THETA system. Although the temperature dependency varies by the antibody and antigen combination, the change of affinity constants is reported to vary ~1- to 20-fold in a physiological temperature range^5,6,31^. The temperature dependency of the equilibrium constant is high in exothermic interactions with large enthalpy changes such as hydrogen bonds and ionic bonds^6^. Therefore, the temperature dependency in the antigen–antibody reaction may mostly be determined by salt bridges formed between the two molecules^6^. Consistent with this, the core epitope in THETAS (TKTKAARMTEQT, underlined amino acids) contains three charged residues, Lys4, Arg7, and Glu10 (Fig. 6b), suggesting that the THETAL:THETAS interaction may be dominated by hydrogen and/or ionic bonds rather than hydrophobic interactions. In general, both the association (*k*_on_) and dissociation (*k*_off_) rate constant for antigen–antibody reactions increase in a temperature-dependent manner, with *k*_off_ having higher temperature dependency than *k*_on_^32^.

In the models of GPD1–GPD6 and THETAS were dissociated at 37 °C as the C13Fv structures were denatured (Fig. 6f). Although this denaturation may be relevant to the temperature dependency of C13Fv, we considered that it was more likely caused by the artificial distortion of the structure during the docking simulation by GalaxyPepDock. Similarly, denaturation was occasionally observed in the other Fv models when docked with the epitope peptide by GalaxyPepDock (Supplementary Fig. 10). In the models of CP1–CP3 (Fig. 6f), the MD simulation and following analysis of changes in RMSD immediately prior to dissociation of C13Fv:THETAS at 37 °C further implied that the temperature-dependent dissociation is triggered by the detachment near the C terminus of THETAS (Fig. 6g). Consistent with this, the Thr9Ala, Gln11Ala, and Thr12Ala mutants showed a lower temperature dependency than the wild-type as evidenced by their affinity constants (Fig. 6b). Notably, these C-terminal three amino acid residues retain polar groups that may form hydrogen bonds with THETAL, as observed in hydrogen bonds between double-stranded nucleotides.

Based on the above considerations, we hypothesized a model for THETAL:THETAS interaction, in which it is composed of the central core region for specific interaction and the adjacent terminal polar region for the thermal interaction with lower specificity. This model suggested that the temperature dependency constituted an intrinsic characteristic of THETAS and that the major determinant of the temperature dependency would be present in THETAS rather than THETAL, although the actual structure of C13Fv:THETAS is likely difficult to be determined owing to its deformability. This is consistent with a previous report for a temperature-dependent antigen–antibody interaction wherein their binding state changed along with the temperature-dependent conformational change of the antigen^33^. Such a mechanism might be relevant to the linear profiles of affinity constants versus temperature (Figs. 1c and 3), which are likely indicative of the lack of temperature-dependent phase transitions in THETAS (or THETAL).

The present study demonstrated the development and first application of the THETA system to our knowledge. Higher temperature dependency might be achieved by using tandem repeats of THETAL and/or THETAS with linkers in appropriate lengths. In silico screening by MD simulation of a systematic series of THETAL:THETAS mutants and in vitro biochemical analyses of thermal dynamics of the selected mutants would provide further clues toward modifying the characteristics of the THETA system. The present study might also have clinical relevance in relation to cold agglutinin disease, a disorder associated with cold-reacting autoantibody-mediated hemolytic anemia^34^. Cold agglutinin disease is so rare that the identity of the cold-reacting antibody is poorly understood. Therefore, further analyses of the THETA system along with other temperature-dependent systems may not only help to establish a novel design principle to create temperature-sensitive protein elements but also provide insight regarding the cold-reacting autoantibodies found in patients with cold agglutinin disease.

## Methods

### Ethics statement

Experiments were conducted in accordance with the guidelines of WASEDA University, and the experimental protocols were approved by the Committee for the Management of Biological Experiment at WASEDA University (permission # 2011-A073, 2012-A052, 2012-A055, WD11-84, WD12-079, WD13-027, WD14-002, WD15-060, WD16-056, WD17-063, and WD18-140).

### Preparation of anti-cCRY4 monoclonal antibody

GST-fusion or MBP-fusion cCRY4CCE (Asp470–Thr529 of chicken CRY4) was used as the antigen for immunization of BALB/c mice^8^. Spleen cells of immunized mice were fused with P3U1 myeloma to form hybridomas. The cells were screened by ELISA using the fusion protein that was not used for immunization. The mAbs were obtained from ascites or culture supernatants of each hybridoma. Epitopes of each monoclonal antibody were determined by competitive ELISA using synthetic peptides for cCRY4CCE (CT1–CT9, Supplementary Fig. 1a).

### Western blot analysis

Immunoblot analysis was performed as described previously^8^. Samples were subjected to SDS-polyacrylamide (10 or 13%) gel electrophoresis, followed by electroblotting onto a polyvinylidene fluoride membrane (Immobilon-P, Millipore). The membrane was incubated for 1 h in SM/TBS (1% (w/v) skim milk (BD Difco), 50 mM Tris-HCl (pH 7.4), 200 mM NaCl, and 1 mM MgCl_2_), and incubated with an antibody diluted in SM/TBS for 3–24 h. Subsequently, the membrane was washed with SM/TBS and incubated with a secondary antibody (1:1000 dilution, alkaline phosphatase-linked anti-mouse IgG, New England Biolabs) in SM/TBS for 1 h. Signals were detected using CDP-*Star* (Roche) and Las-1000 (GE Healthcare).

### Measurement of affinity constants by indirect competitive ELISA

Affinity constant between monoclonal antibody and each antigen was measured by indirect competitive ELISA^35^. Briefly, Nunc-Immuno plates (Thermo Scientific) were coated with GST-cCRY4CCE diluted in TBS (50 mM Tris-HCl (pH 7.4), 200 mM NaCl, and 1 mM MgCl_2_) to 1 μg mL^−1^ or 0.1 μg mL^−1^. An antibody diluted with SM/TBS was mixed with serially diluted antigens and incubated until the equilibrium state. Then, the mixture was transferred to a well that had been coated with GST-cCRY4CCE and blocked with SM/TBS, and then incubated for 15 min. Concentrations of the coated antigen were determined by ELISA in advance: 1 μg mL^−1^ for C13 mAb and 0.1 μg mL^−1^ for C14 and C15 antibodies. The binding signals were detected with a secondary antibody (anti-mouse IgG (H + L) Antibody Human Serum Adsorbed and Peroxidase labeled, KPL) and TMB solution (50 mM citrate, 100 mM Na-Pi (pH 5.0), 0.01% (w/v) 3,3′, 5,5′-tetramethylbenzidine (Sigma), 1% (v/v) DMSO and 0.006% (v/v) H_2_O_2_). Differential absorbance at 450 nm and 620 nm was measured with a plate reader (Bio-Rad, Model 680). By using solver in Excel, affinity constants were determined by fitting the relationship between antigen concentration and absorbance to a model formula^36^.

### Preparation of CT9 fusion proteins

For immunoaffinity chromatography, one to three copies of CT9 and D2 peptide (EFSHRGSQRN) were added to the N-terminal side or C-terminal side. The D2 peptide is an epitope of the D2 monoclonal antibody that we had previously produced. For expression of the fusion proteins with the N-terminal or C-terminal tag, pDEST24 (Thermo Fisher Scientific) or pGEX-5X-1 (GE Healthcare) was used, respectively. BL21 (DE3) was transformed with the expression vector.

### Cloning and construction of C13scFv

Complementary DNAs encoding the heavy and light chains of C13 mAb were isolated from a cDNA pool of C13 hybridoma by PCR using degenerated primers modified from Wang et al.^37^ as follows: heavy-chain constant region, heavy-chain FR1 region (MH1), kappa chain-constant region, and kappa-chain FR1 region (Table 1). Then, the genes of C13 variable light (VL), (GGGGS)_3_ linker, and C13 variable heavy (VH) were amplified by PCR using primers as follows: C13_VL_for, C13_VL_rev, linker_for, linker_rev, C13_VH_for, C13_VH_rev (Table 1), and their amplified genes and the linearized pET-22b (+) plasmid were fused using the In-Fusion HD cloning kit (TaKaRa). Accession number of C13scFv sequence is LC461992. The *MBP* gene was amplified by PCR using pMAL-c2 as a template (primers: In_fusion_MBP_for, In_fusion_MBP_rev) (Table 1), and the amplified *MBP* gene was ligated with the linearized pET-22b (+) plasmid by using the In-Fusion HD cloning kit (TaKaRa). BL21 (DE3) was transformed with the completed construct.

**Table 1:** Sequence of primers using cloning and construction of C13scFv

Name	Sequence
Heavy chain constant region	5′-GTG TAC AAT AGA CAG ATG GGG GTG TCG T-3′
Heavy chain FR1 region (MH1)	5′-GTC GAC SAR GTN MAG CTG SAG SAG TC-3′
Kappa chain constant region	5′-GGT ACC GGA TAC AGT TGG TGC AGC ATC-3′
Kappa chain FR1 region	5′-CTC GAG GAY ATT GTG MTS ACM CAR WC-3′
C13_VL_for	5′-CCG GCG ATG GCC ATG GAC ATT GTG ATG ACC CAA TCT CAC A-3′
C13_VL_rev	5′-GGA TAC AGT TGG TGC AGC ATC AGC C-3′
Linker_for	5′-GCA CCA ACT GTA TCC GGA GGC GGT GGT TCA GGT GG-3′
Linker_rev	5′-CTC CAG CTT AAC CTC AGA ACC TCC TCC GCC CGA TC-3′
C13_VH_for	5′-GAG GTT AAG CTG GAG GAG TCT GGA G-3′
C13_VH_rev	5′-GCT CGA ATT CGG ATC ATA GAC AGA TGG GGG TGT CGT TTT G-3′
In_fusion_MBP_for	5′-CCA GCC GGC GAT GGC CCC ATG GAT GAA AAT CGA AGA AGG TAA ACT GGT AAT CTG G-3′
In_fusion_MBP_rev	5′-ATC ACA ATG TCC ATG CCC CTT CCC TCG ATC CCG AGG T-3′

### Purification and refolding of MBP-THETAL-His

BL21 (DE3) expressing MBP-THETAL-His was suspended in *E. coli* breaking buffer (50 mM Tris-HCl (pH 7.5), 100 mM NaCl, 1 mM EDTA, 2 mM PMSF, 0.5 mg L^−1^ aprotinin, 0.1 mg L^−1^ pepstatin, and 1 mg L^−1^ leupeptin) and broken by sonication (Sonifier 150, BRANSON). The cell suspension was centrifuged (4 °C, 22,140 × *g*, 30 min) to remove the supernatant. The precipitate was washed once with pellet wash buffer (50 mM Tris-HCl (pH 7.5), 500 mM NaCl, 1 mM EDTA, 5% (v/v) Triton X-100, 5% (v/v) Tween 20, 5% (v/v) NP-40, 5 mM DTT), and once with 50 mM Tris-HCl (pH 7.5). Then, MBP-THETAL-His was extracted from the precipitate by treatment with denaturing buffer (50 mM Tris-HCl (pH 7.5), 8 M urea) for 1 h at room temperature. Next, the extract was gradually diluted with 14 volumes of refolding buffer (55 mM Tris-HCl (pH 10.7), 21 mM NaCl, 1 mM EDTA, 0.88 mM KCl, 2 mM reduced glutathione, 0.2 mM oxidized glutathione, and 440mM l-arginine) and stirred overnight (4 °C and 10 rpm). The diluted extract was further concentrated by the Amicon Model 8200 stirred cell using a YM 10 membrane (Millipore) and dialyzed against PBS (10 mM Na-Pi (pH7.4) and 140 mM NaCl).

### Thermal-elution-based purification using C13-mAb- or THETAL-immobilized gel

C13 mAb and the renatured MBP-THETAL-His were immobilized on Affi-Gel 10 and Affi-Gel 15, respectively (Bio-Rad). The crude *E. coli* extract or chick retinal soluble fraction was mixed with the immunoaffinity gel and rotated at 4 °C. After the reaction, unbound materials were removed and the gel was loaded on the cooled jacketed column with wash buffer (50 mM Tris-HCl (pH 7.5) and 100 mM NaCl). After washing the column with wash buffer, the column buffer was changed to elution buffer (50 mM Tris-HCl (pH 7.5), 100 mM NaCl, and 50% (v/v) glycerol) to collect a few fractions at 4 °C. Then, the temperature of the column was raised by circulating 42 °C or 37 °C water in the column jacket for 10–30 min. The bound materials were eluted with elution buffer at 42 °C or 37 °C.

### Thermal-elution-based purification of THETAL by biotinylated THETAS-immobilized gel

N-terminally bio-THETAS was immobilized to streptavidin sepharose (GE Healthcare) in binding buffer (20 mM Na-Pi (pH 7.5) and 150 mM NaCl). A crude cell extract of BL21(DE3) expressing THETAL-His was added to the bio-THETAS-immobilized-gel at 4 °C. After washing with binding buffer at 4 °C, THETAL-His was eluted with binding buffer at 37 °C.

### Structure prediction of C13Fv and THETAS

The structure of C13Fv was predicted from the sequence information of the C13 antibody variable region (Fig. 4a) by using ABodyBuilder^18^, Kotai Antibody Builder^19^, and PIGSPro^20^. PDB:2AEP was selected as the template for light chain and heavy chain and PDB:4KZE as the template for H3 in the prediction by ABodyBuilder. Kotai Antibody Builder built the structure selecting PDB:2AEP as the template for light chain and heavy chain and PDB:3CX5J as the template for H3. PIGSPro built the structure according to PDB:5F3B as the template for light chain, PDB:3DUU as the template for heavy chain, and PDB:4PB9 as the template for H3. The structure of THETAS was predicted using PEP-FOLD 3.5^21^ and PEPstrMOD^22^. In PEP-FOLD, three structures of the top three scores were chosen. In PEPstrMOD, three structures were predicted under each environmental condition of vacuum, hydrophilic, and hydrophobic. PyMOL 2.1.0 was used for rendering of the molecular structures.

### Docking simulation of C13Fv and THETAS

ClusPro 2.0^23^, CABS-dock^25^, GalaxyPepDock^26^, and pepATTRACT 2.0^27^ were used for docking simulations of C13Fv and THETAS. In ClusPro, the predicted structures of C13Fv and THETAS were combinatorially docked in Antibody Mode^24^. In CABS-dock, GalaxyPepDock, and pepATTRACT, the predicted structures of C13Fv and the amino acid sequence of THETAS were used for simulation. The interaction sites in the docked complex were analyzed by LigPlot + ^38^.

### MD simulation

MD simulations were performed by using NAMD 2.12 Win 64-CUDA^39^ based on the force field CHARMM22, in which water molecules were added and charge was minimized. The target temperature was set at 277 K or 310 K. RMSD of the α carbon of each amino acid was analyzed with the RMSD Visualizer Tool of VMD 1.9.3^40^.

### Reporting summary

Further information on research design is available in the Nature Research Reporting Summary linked to this article.

## Supplementary information

Supplementary Information

Description of additional supplementary items

Supplementary Movie 1

Supplementary Movie 2

Supplementary Movie 3

Reporting Summary

## Data Availability

The data supporting the findings of this study are available within the paper and its Supplementary information files. MD simulation results of CP1, CP2, and CP3 are provided in Supplementary Movies 1–3. Full blots used for western blot analysis are shown in Supplementary Fig. 2.
