# Supplementary material for: THETA system allows one-step isolation of tagged proteins through temperature-dependent protein–peptide interaction
Source: Commun Biol. 2019 Jun 14;2:207. doi: 10.1038/s42003-019-0457-8 (PMC6572768; doi:10.1038/s42003-019-0457-8)
Supplement: Supplementary file 1 — Supplementary Information [file 42003_2019_457_MOESM1_ESM.pdf]

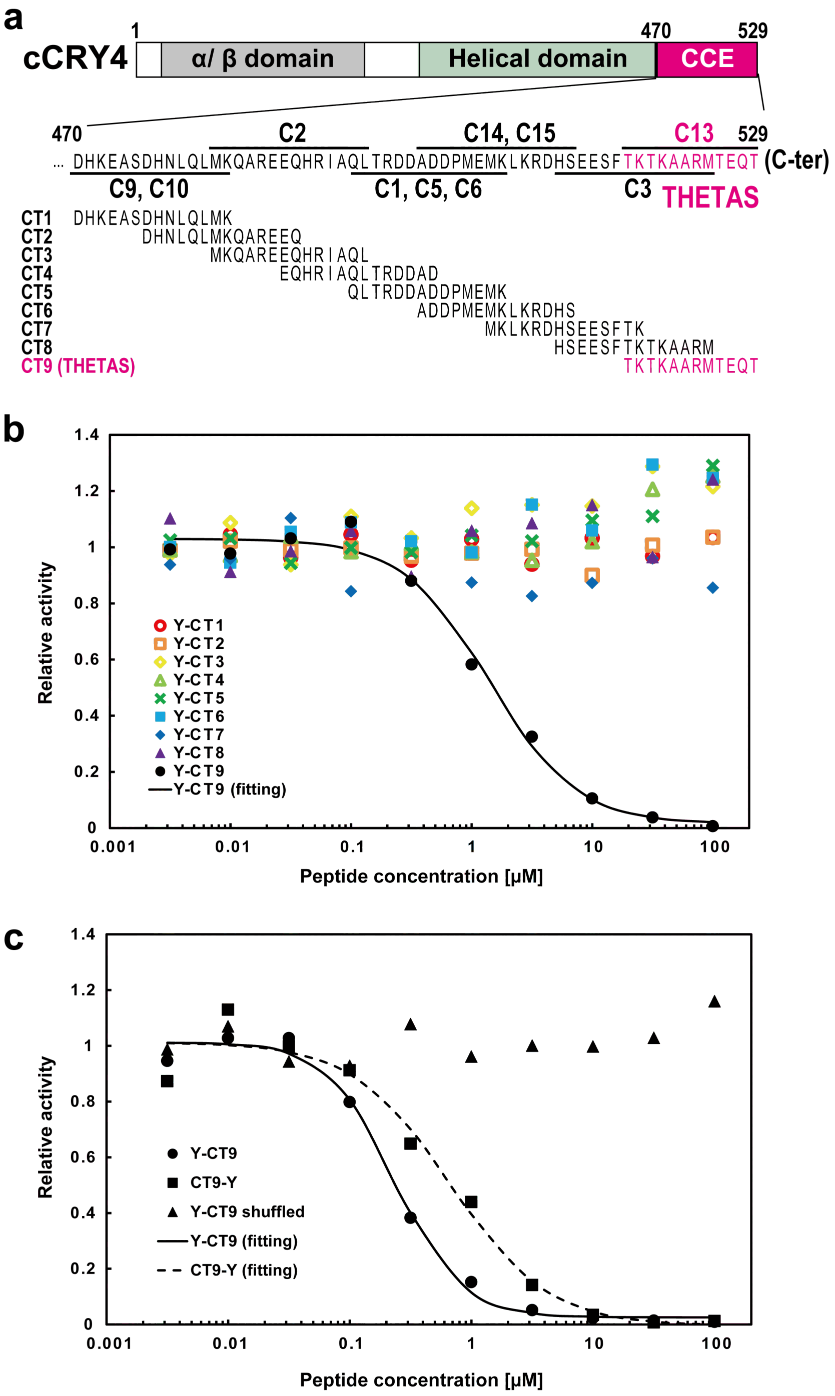

Supplementary Figure 1. Epitope analysis of C13 mAb by competitive ELISA.
(**a**) cCRY4 C-terminal extension (CCE) sequence and epitope locations for 10 monoclonal antibodies. Epitopes for 10 out of the 15 antibodies were roughly localized by competitive ELISA using synthetic short peptides CT1 to CT9 into which the CCE region of cCRY4 was divided. (**b**) C13 mAb was mixed with serially diluted peptides (N-terminally tyrosinated CT1–CT9) and incubated for 1 h at 37 °C. (**c**) C13 mAb was mixed with serially diluted N-terminally or C-terminally tyrosinated CT9 peptides (Y-CT9 [YTKTKAARMTEQT] or CT9-Y [TKTKAARMTEQTY]) or N-terminally tyrosinated CT9 shuffled peptide (Y-CT9 shuffled; YTAKATMKTRQET) and incubated for 1 h at 4 °C. They were transferred to a well that had been coated with GST-cCRY4CCE and blocked with SM/TBS, then incubated for 1 h. The binding signals were detected with a secondary antibody and TMB solution, and were measured as differential absorbance at 450 nm and 620 nm. The signals were normalized with the averages of the bottom three points. Fitting curves were calculated by the Rodbard method using ImageJ.

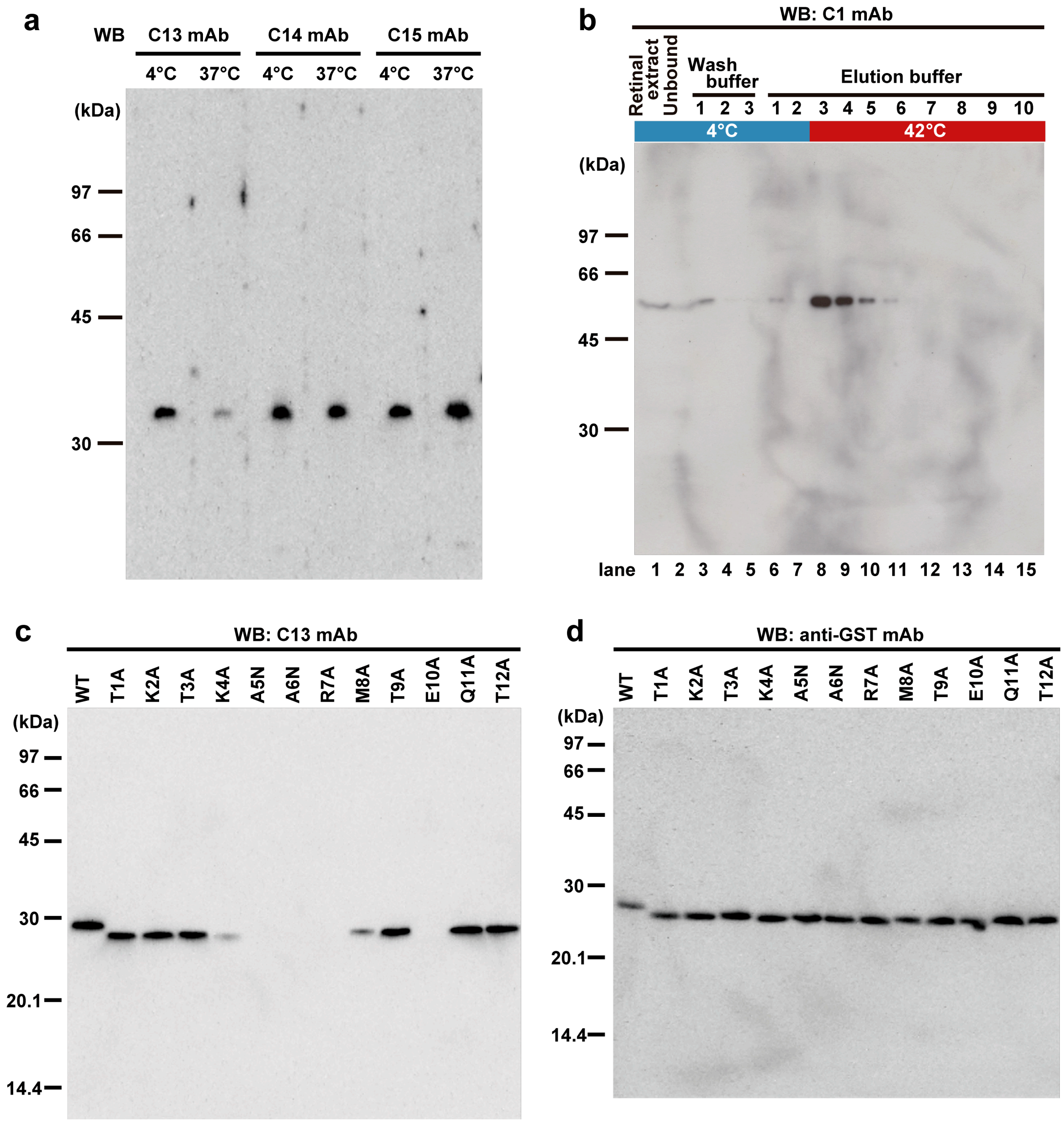

Supplementary Figure 2. Overall picture of each western blot analysis.
(**a**) Fig. 1b was excerpted from a part of this figure. (**b**) Fig. 2b was excerpted from a part of this figure. (**c**, **d**) Fig. 6c was excerpted from a part of this figure.

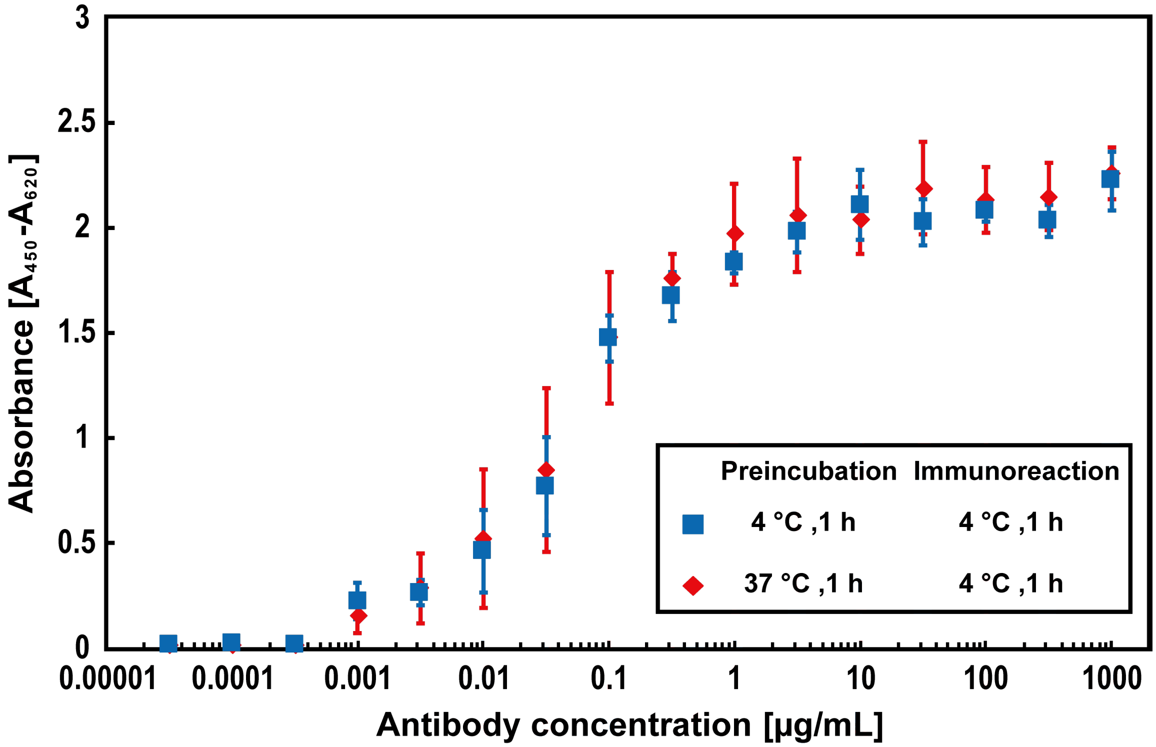

Supplementary Figure 3. Immunoreactivity of C13 mAb is not affected by pre-incubation at 37 °C. C13 mAb, which had been pre-incubated for 1 h at 4 °C or 37 °C, was transferred to a well that had been coated with GST-cCRY4CCE and blocked with SM/TBS, then incubated for 1 h at 4 °C. The binding signals were detected with a secondary antibody and TMB solution, and measured as differential absorbance at 450 nm and 620 nm. Error bars represent standard deviation (n = 3).

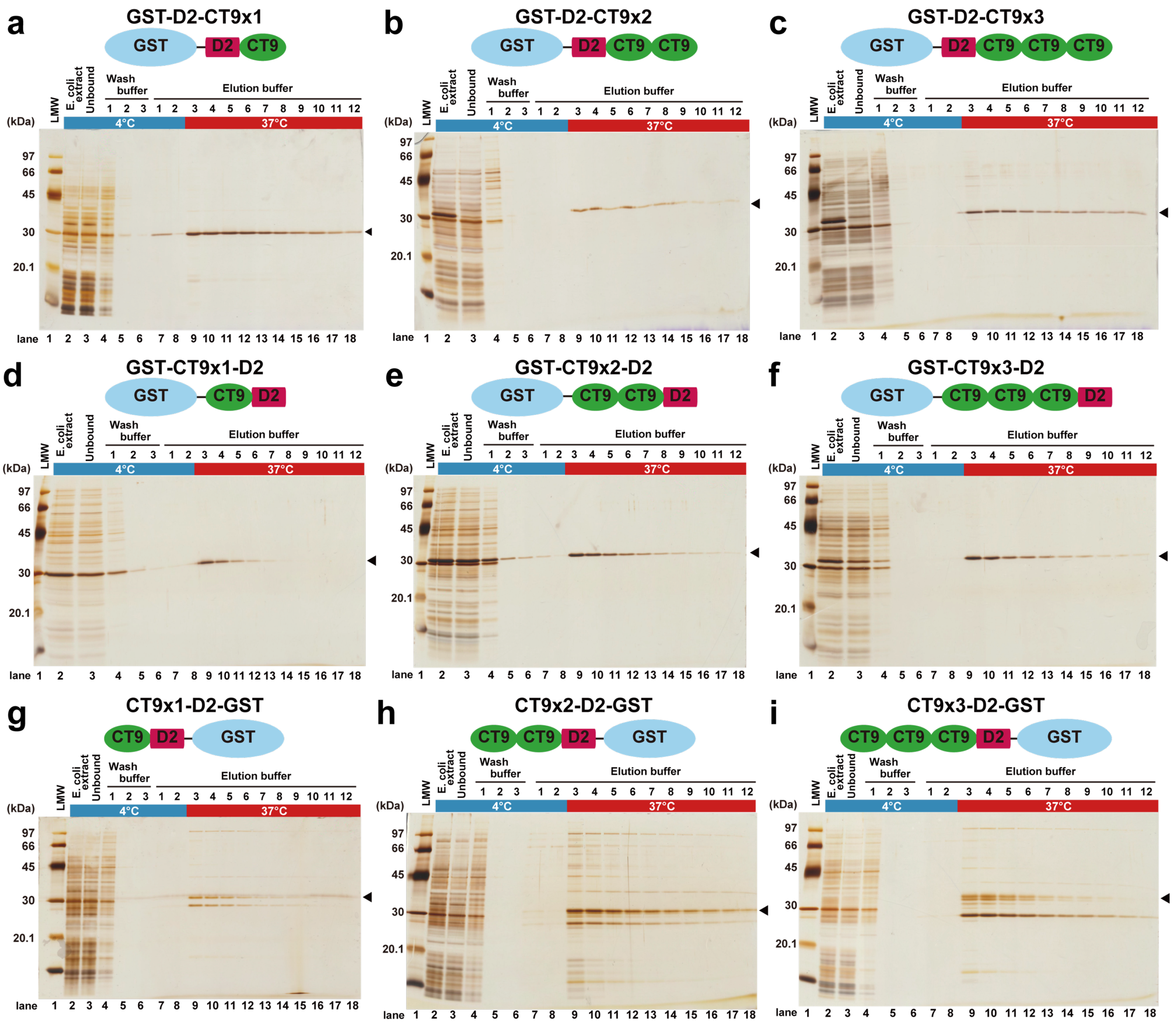

Supplementary Figure 4. Affinity purification of recombinant CT9 fusion proteins by thermal elution from C13-mAb-gel.
The cell extracts from *E. coli* expressing the various CT9 fusion proteins were applied to 1 mL of C13-mAb-gel, and the gel was washed 3 times with 5 mL of wash buffer at 4 °C, followed by successive treatments with 1 mL of elution buffer twice at 4 °C and 10 times at 42 °C. The column fractions were analyzed by SDS-PAGE/silver staining. Fig. 3 was excerpted from a part of this figure.

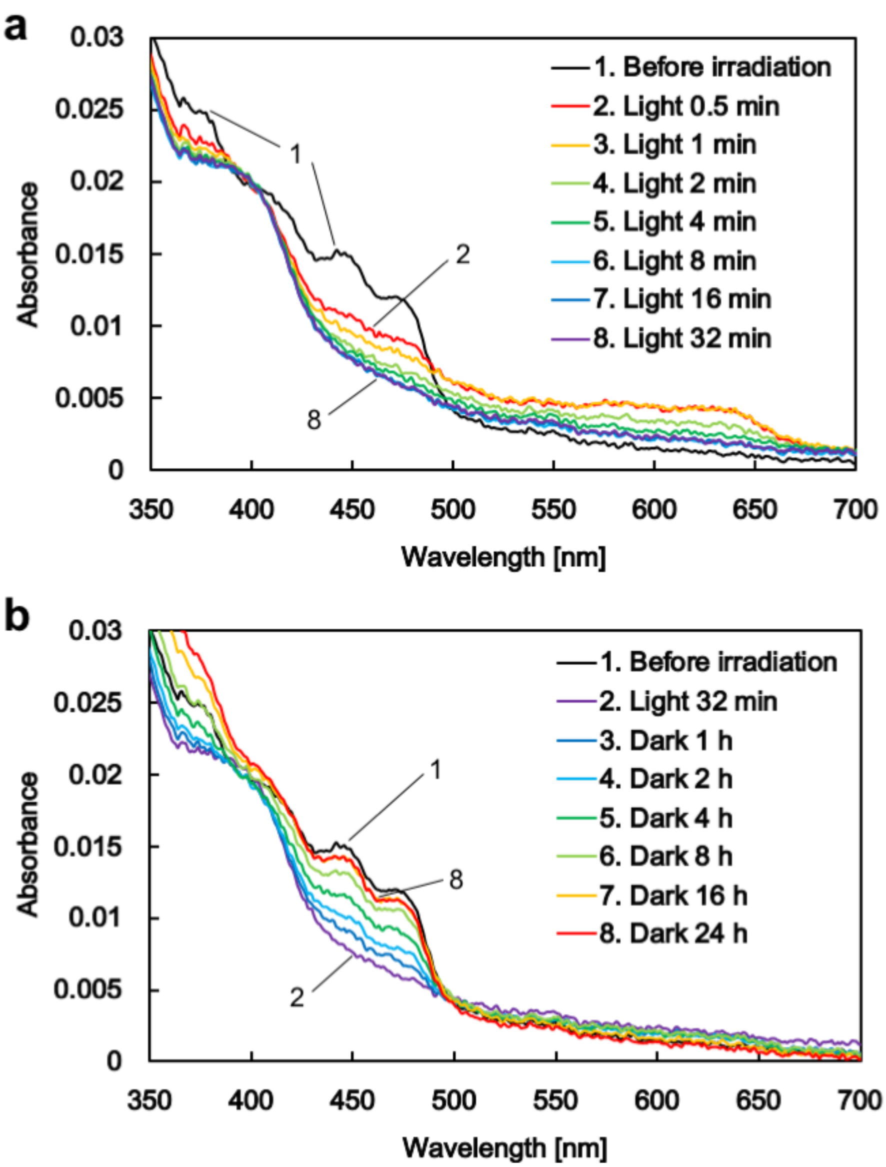

Supplementary Figure 5. Ultraviolet-visible spectroscopic analysis of cCRY4 purified by using MBP-THETAL-His-gel.

Ultraviolet-visible spectroscopic analysis was carried out as described previously (1). (**a**) Photoreduction of FAD chromophore by blue light irradiation. Absorption bands at 350 to 500 nm originating from fully oxidized FAD were observed in the initial dark state (curve1, Before irradiation). When irradiated with blue light for 0.5 min (λ_{max} = 453 nm, 1 mW cm^{-2}), the absorption decreased at 350 to 500 nm and increased at 500 to 650 nm (curve2, Light 0.5 min) as indicative of formation of the neural radical form of FAD (FADH•). Further irradiation induced the absorbance decrease at 350 to 500 nm (curve 3) and later at a wide wavelength range of 350 to 700 nm (curves 4–8) showing photoreduction of FAD to the fully reduced form via the FADH•. (**b**) Dark oxidization of the chromophore FAD after photoreduction by the blue light. After blue light irradiation for 32 min (curve 2), the sample was incubated in the dark at 20 °C for 24 h (curves 3–8). Curve 1, initial dark state prior to the irradiation.

	L chain			L1									L2												
	414	415	...	440	441	442	443	444	445	446	447	...	458	459	460	461	462	463	464	465	466	467	468	469	...
	D	I	...	Q	D	V	G	T	A	V	A	...	K	L	L	I	Y	W	A	S	T	R	H	T	...
CP1																									
CP2																									
CP3								*										*							
GPD1																									
GPD2					*	*	*	*								*	*								
GPD3																									
GPD4								*		*			(A6)			(A5)	*					*		*	
GPD5																	*								
GPD6						*	*										*								

				L3									
...	500	501	502	503	504	505	506	507	508	509	...	528	529
...	F	C	Q	Q	Y	S	S	Y	P	Y	...	V	S
					R7	*	*	T3		(A6)				CP1
					*									CP2
					*	K4	*	*		*				CP3
			*		*			*		*				GPD1
						T9		*						GPD2
					*	(A6)		(K4)		K4				GPD3
					T9	*				R7				GPD4
					*					*				GPD5
														GPD6

	H chain			H1										H2																	
	545	546	...	571	572	573	574	575	576	577	578	579	...	591	592	593	594	595	596	597	598	599	600	601	602	603	604	605	606	...
	E	V	...	F	T	F	T	D	Y	Y	M	S	...	W	L	G	F	I	R	N	K	A	N	V	Y	T	T	E	Y	...
CP1								(T12)		(M8)							*		(K4)	(E10)	*	*	*				*	(K4)		
CP2								T3,R7	(T1)	T12							*		T12	(K4)		*								
CP3										*									*			Q11	*							
GPD1								R7	*	(R7)	*	*		*			*		E10	*			(R7)							
GPD2										*									E10				(Q11)							
GPD3								(T1)	*	*									*	(T1)			E10					R7		
GPD4										E10									(E10)											
GPD5										Q11				*			*		*	(Q11)		*	*							
GPD6					*		(T1)	T1												*		*	*							

							H3											
...	643	644	645	646	647	648	649	650	651	652	653	654	655	656	657	...	676	677
...	A	R	D	G	G	Y	D	D	G	G	Y	A	M	D	Y	...	V	Y
						*					(T9)								CP1
			*	K4	*	Q11	*	K2		K4	Q11								CP2
							*	R7		*									CP3
	Q11	*	T9			*	(K4)	K4		*	*		Q11						GPD1
						R7	K4	K4	*	*	(E10)								GPD2
			K4		*		K2	K2	*		K4		*						GPD3
			*	R7	*	*	K2	*	*	*	(T1)	(A6)		*					GPD4
			R7		T12	*	T3	(T12)	*	(K4)	(K4)								GPD5
				*	(A5)	*	*	K4	*	*	*			R7					GPD6

Supplementary Figure 6. Amino acid residues of C13Fv interacting with THETAS in docked structures.

Amino acid residues in each of the nine selected structures (three from ClusPro [CP1–CP3] and six from GalaxyPepDock [GPD1–GPD6]) were examined for the potential to form hydrogen bonds to any residue in THETAS by using LigPlot+ (2). The amino acid within THETAS forming a hydrogen bond is indicated with the residue number under the amino acid position of C13Fv. Amino acids in parentheses denote that atoms in the main chain are involved in the hydrogen bond. The amino acids that were considered to be important from in vitro epitope analysis are highlighted in yellow. Asterisks indicate involvement in hydrophobic interaction with THETAS.

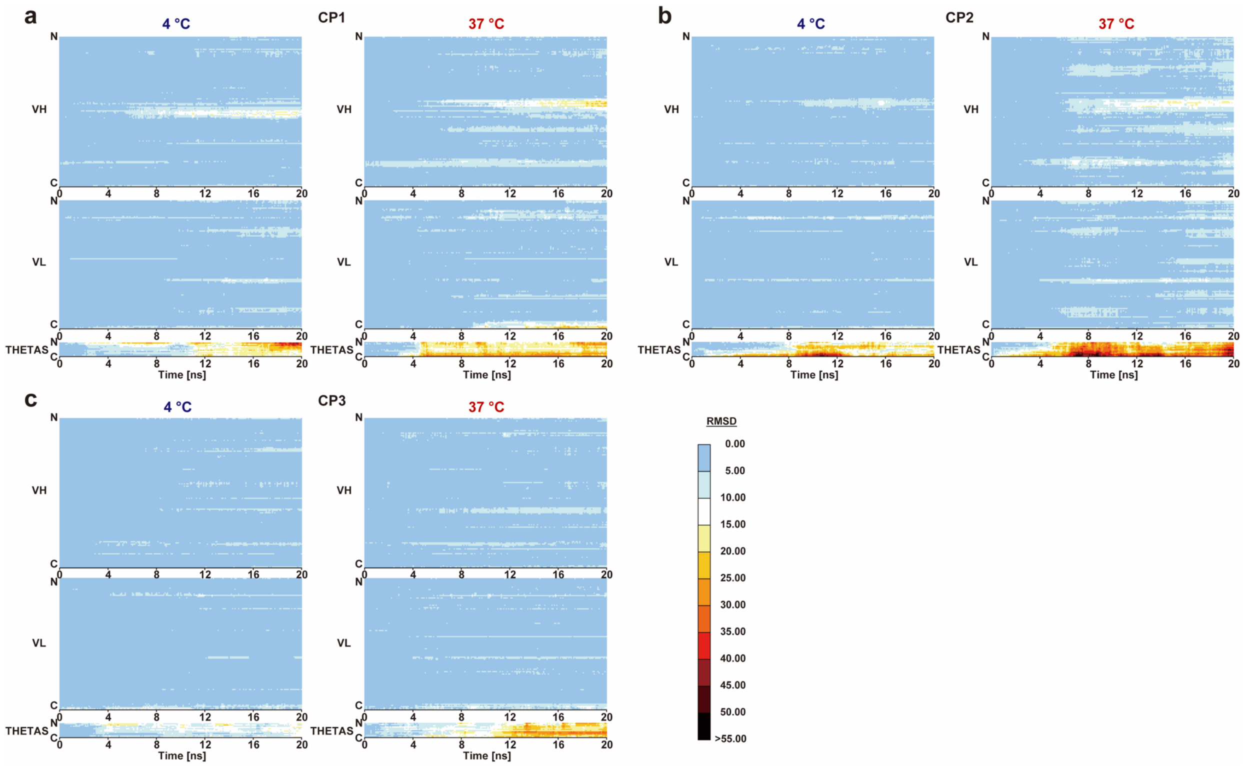

Supplementary Figure 7. RMSD of C13Fv:THETAS structure docked by ClusPro.
MD simulations were performed by 20 ns at 4 °C or 37 °C using three selected structures (CP1–CP3) generated by ClusPro (3). RMSD of the α-carbon of each amino acid from the starting structure was calculated and plotted with RMSD visualizer tool, a plugin of VMD.

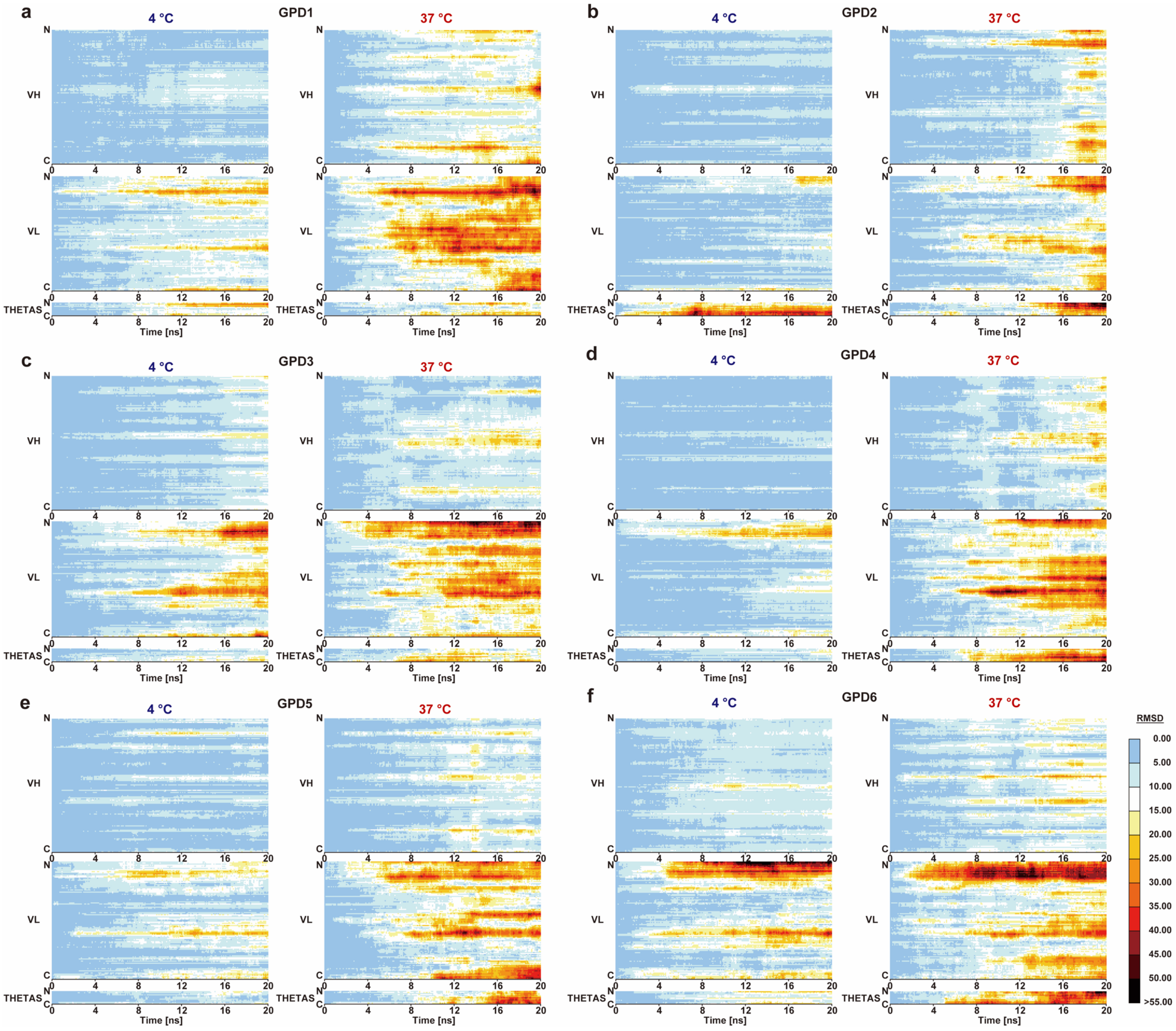

Supplementary Figure 8. RMSD of C13Fv:THETAS structure docked by GalaxyPepDock.
MD simulations were performed by 20 ns at 4 °C or 37 °C using six selected structures (GPD1–GPD6) generated by GalaxyPepDock (4). RMSD of the α-carbon of each amino acid from the starting structure was calculated and plotted with RMSD visualizer tool.

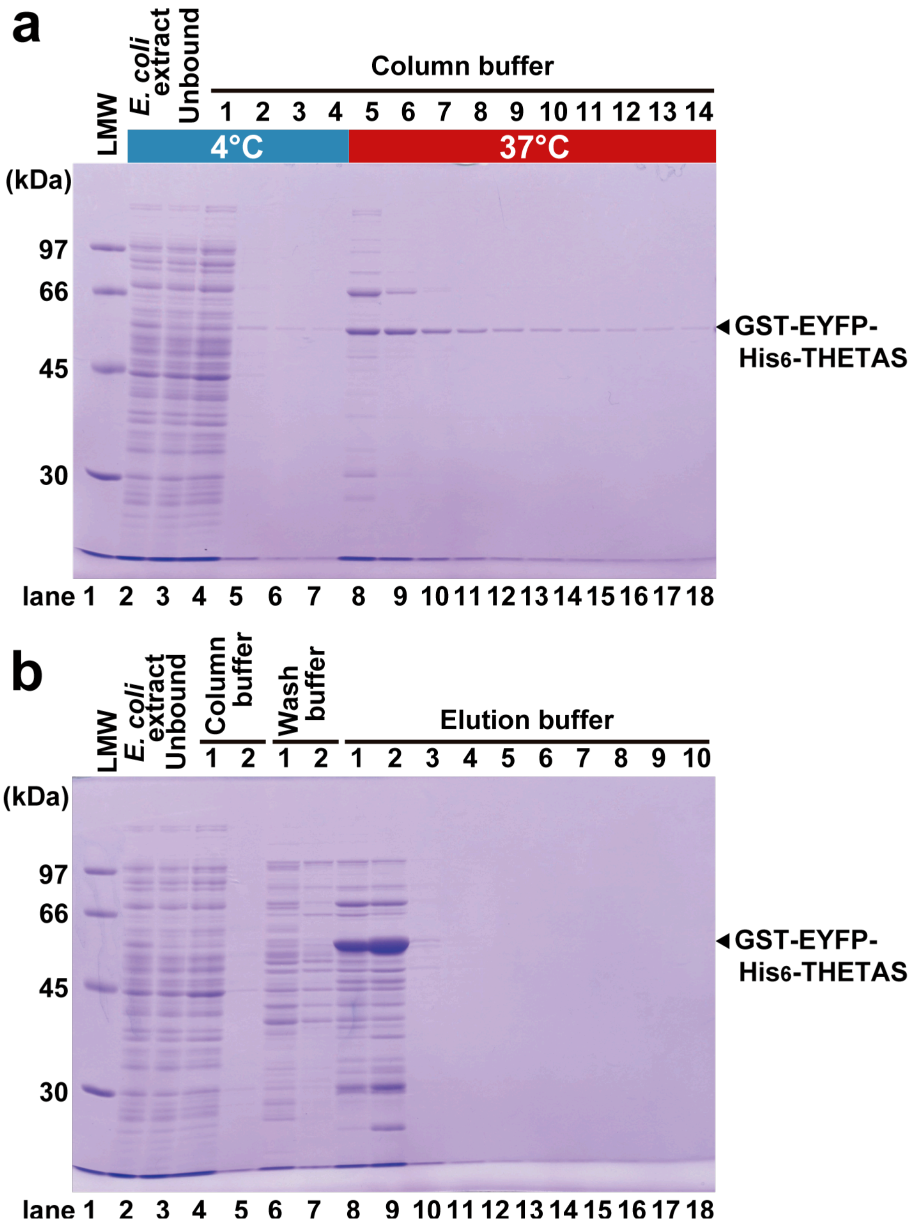

Supplementary Figure 9. Affinity purification of GST-EYFP-His$_6$-THETAS by THETA system and Ni-NTA system.

a Affinity purification of GST-EYFP-His$_6$-THETAS by THETA system. The crude *E.coli* soluble extracts were applied to 2 mL of MBP-THETAL-His-immobilized gel, and the gel was washed 4 times with 5 mL of Column buffer (50 mM Tris-HCl (pH 7.5), 100 mM NaCl) at 4 °C. Then, the temperature of the column was raised by circulating 37 °C water in the column jacket for 10 min. The bound materials were eluted 10 times with Column buffer at 37 °C. The column fractions were analyzed by SDS-PAGE/Coomasie brilliant blue staining.

b Affinity purification of GST-EYFP-His$_6$-THETAS by Ni-NTA system. The crude *E.coli* soluble extracts were applied to 2 mL of Ni Sepharose 6 Fast Flow (GE Healthcare), and the gel was washed 2 times with 5 mL of Column buffer (50 mM Tris-HCl (pH 7.5), 100 mM NaCl) and 2 times with 5 mL of Wash buffer (50 mM Tris-HCl (pH 7.5), 100 mM NaCl, 20 mM Imidazole). The bound materials were eluted 10 times with Elution buffer (50 mM Tris-HCl (pH 7.5), 100 mM NaCl, 500 mM Imidazole). The column fractions were analyzed by SDS-PAGE/Coomasie brilliant blue staining. The calculated molecular mass of GST-EYFP-His$_6$-THETAS is 56,232 Da.

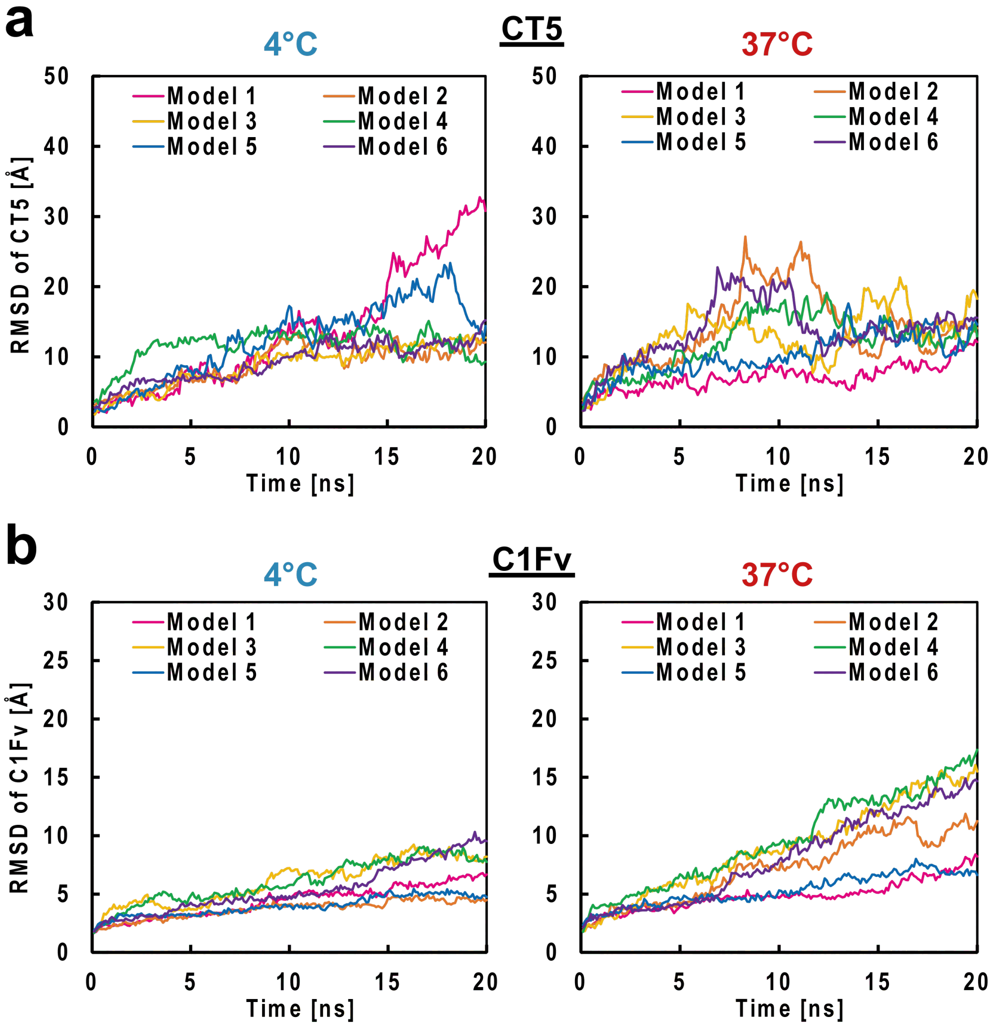

Supplementary Figure 10. Average value of RMSD of C1Fv:CT5 structure.
The structure of Fv of the C1 mAb (C1Fv) was predicted using ABodyBuilder (5), Kotai Antibody Builder (6), and PIGSPro (7). Then, the predicted structures of C1Fv together with the amino acid sequence of its epitope-containing peptide CT5 (QLTRDDADDPMEMK) were subjected to docking simulation by GalaxyPepDock (4). A total of six predicted structures of C1Fv (Model 1 to Model 6, 1st place or 2nd place with Estimated accuracy score in each of the three predicted methods) were used as the starting structures of MD simulation. Temporal changes in averaged RMSDs of CT5 (**a**) or C1Fv (**b**) in the MD simulation.

Supplementary Table 1. Affinity constants for C13-C15 anti-cCRY4CCE mAbs against GST-cCRY4CCE or GST-CT9.

Antibody	Antigen	Temperature [°C]	Affinity constant ± S.D. [$\times 10^6$ M^{-1}]		
C13 mAb	GST-cCRY4CCE	4	527.5	±	148.8
		15	238.4	±	23.40
		26	37.85	±	5.978
		37	4.317	±	0.9452
C14 mAb	GST-cCRY4CCE	4	775.5	±	129.2
		15	506.3	±	52.98
		26	691.8	±	91.30
		37	908.5	±	244.0
C15 mAb	GST-cCRY4CCE	4	1018	±	316.1
		15	534.9	±	106.4
		26	798.9	±	21.48
		37	1130	±	482.1
C13 mAb	GST-CT9	4	3654	±	451.1
		15	1086	±	350.8
		26	18.69	±	2.821
		37	7.662	±	0.2504

Supplementary Table 2. Affinity constants of C13 mAb and CT9 fusion proteins

Antigen	Temperature [°C]	Affinity constant ± S.D. [×10^{6} M^{-1}]
GST-D2-CT9x1	4	1234 ± 175.1
	15	612.6 ± 155.8
	26	96.01 ± 16.10
	37	10.31 ± 1.399
GST-D2-CT9x2	4	1276 ± 181.3
	15	562.9 ± 48.48
	26	143.1 ± 36.81
	37	20.44 ± 2.139
GST-D2-CT9x3	4	1763 ± 70.22
	15	640.2 ± 58.02
	26	116.7 ± 4.633
	37	24.80 ± 1.521
GST-CT9x1-D2	4	133.1 ± 16.48
	15	62.47 ± 5.829
	26	4.943 ± 0.6239
	37	3.839 ± 0.9062
GST-CT9x2-D2	4	372.8 ± 32.96
	15	50.84 ± 5.680
	26	10.10 ± 1.005
	37	2.014 ± 0.3128
GST-CT9x3-D2	4	1849 ± 140.3
	15	486.0 ± 45.18
	26	164.9 ± 42.78
	37	6.488 ± 1.039
CT9x1-D2-GST	4	101.3 ± 12.61
	15	12.38 ± 1.491
	26	4.576 ± 1.373
	37	2.313 ± 0.2053
CT9x2-D2-GST	4	1250 ± 268.8
	15	258.6 ± 57.55
	26	43.59 ± 8.013
	37	4.405 ± 1.151

Supplementary Table 3. Affinity constants of C13 mAb and mutated THETAS

Antigen	Temperature [°C]	Affinity constant ± S.D. [×10^6 M^{-1}]		
GST-THETAS_Thr1Ala	4	2976	±	1051
	15	885.8	±	166.2
	26	27.70	±	1.966
	37	9.215	±	1.737
GST-THETAS_Lys2Ala	4	1265	±	68.34
	15	294.3	±	83.71
	26	26.46	±	3.613
	37	11.79	±	2.678
GST-THETAS_Thr3Ala	4	1715	±	275.5
	15	328.0	±	18.70
	26	21.12	±	4.099
	37	10.45	±	2.189
GST-THETAS_Lys4Ala	4	103.1	±	13.19
	15	10.35	±	3.465
	26	2.153	±	0.2615
	37	1.133	±	0.1124
GST-THETAS_Met8Ala	4	207.3	±	15.69
	15	25.89	±	4.820
	26	4.592	±	0.6800
	37	3.983	±	2.351
GST-THETAS_Thr9Ala	4	2366	±	470.8
	15	445.7	±	41.59
	26	24.18	±	3.497
	37	35.57	±	12.93
GST-THETAS_Gln11Ala	4	2948	±	373.6
	15	1258	±	77.13
	26	61.95	±	18.87
	37	56.03	±	22.82
GST-THETAS_Thr12Ala	4	3450	±	531.9
	15	1003	±	202.4
	26	47.21	±	10.67
	37	30.90	±	7.993
GST-THETAS (= GST-CT9)	4	3654	±	451.1
	15	1086	±	350.8
	26	18.69	±	2.821
	37	7.662	±	0.2504

Supplementary Table 4. Docking simulation and MD simulation of C13Fv:THETAS

ID	Docking simulation			MD simulation	
	Docking tool	Antibody prediction tool	Antigen prediction tool	Temperature [°C]	Time when THETAS dissociated [ns]
CP1	ClusPro	ABodyBuilder	PEPstrMOD (hydrophilic)	4	17.5
				37	4.8
CP2	ClusPro	ABodyBuilder	PEPstrMOD (vacuum)	4	8.8
				37	5.5
CP3	ClusPro	PIGSPro	PEPstrMOD (hydrophobic)	4	>20
				37	12.4
GPD1	GalaxyPepDock	ABodyBuilder	(A.A. sequence)	4	>20
				37	15.5
GPD2	GalaxyPepDock	ABodyBuilder	(A.A. sequence)	4	6.1
				37	14.6
GPD3	GalaxyPepDock	Kotai Antibody Builder	(A.A. sequence)	4	>20
				37	11.7
GPD4	GalaxyPepDock	PIGSPro	(A.A. sequence)	4	>20
				37	11.2
GPD5	GalaxyPepDock	PIGSPro	(A.A. sequence)	4	>20
				37	15.1
GPD6	GalaxyPepDock	PIGSPro	(A.A. sequence)	4	>20
				37	8.3

Supplementary References

1. Mitsui, H. *et al*. Overexpression in yeast, photocycle, and in vitro structural change of an avian putative magnetoreceptor cryptochrome4. *Biochemistry* **54**, 1908-1917 (2015).
2. Laskowski, R. A. & Swindells, M. B. LigPlot+: multiple ligand-protein interaction diagrams for drug discovery. *J. Chem. Inf. Model.* **51**, 2778-2786 (2011).
3. Kozakov, D. *et al*. The ClusPro web server for protein-protein docking. *Nat. Protoc.* **12**, 255-278 (2017).
4. Lee, H., Heo, L., Lee, M. S. & Seok, C. GalaxyPepDock: a protein-peptide docking tool based on interaction similarity and energy optimization. *Nucleic Acids Res.* **43**, W431-5 (2015).
5. Leem, J., Dunbar, J., Georges, G., Shi, J. & Deane, C. M. ABodyBuilder: Automated antibody structure prediction with data-driven accuracy estimation. *MAbs* **8**, 1259-1268 (2016).
6. Yamashita, K. *et al*. Kotai Antibody Builder: automated high-resolution structural modeling of antibodies. *Bioinformatics* **30**, 3279-3280 (2014).
7. Lepore, R., Olimpieri, P. P., Messih, M. A. & Tramontano, A. PIGSPro: prediction of immunoGlobulin structures v2. *Nucleic Acids Res.* **45**, W17-W23 (2017).
