# Supplementary material for: THETA system allows one-step isolation of tagged proteins through temperature-dependent protein–peptide interaction
Source: Commun Biol. 2019 Jun 14;2:207. doi: 10.1038/s42003-019-0457-8 (PMC6572768; doi:10.1038/s42003-019-0457-8)
Supplement: Supplementary file 2 — Description of additional supplementary items [file 42003_2019_457_MOESM2_ESM.docx]

**Supplementary Movie 1. MD simulation of the model of CP1.**

**Supplementary Movie 2. MD simulation of the model of CP2.**

**Supplementary Movie 3. MD simulation of the model of CP3.**

These movies are the results of MD simulation of each structure. MD simulations were performed by 20 ns

at 277 K or 310 K. When RMSD of THETAS exceeded 20 Å, the frame color changes to red.
