# Supplementary material for: THETA system allows one-step isolation of tagged proteins through temperature-dependent protein–peptide interaction
Source: Commun Biol. 2019 Jun 14;2:207. doi: 10.1038/s42003-019-0457-8 (PMC6572768; doi:10.1038/s42003-019-0457-8)
Supplement: Supplementary file 6 — Reporting Summary [file 42003_2019_457_MOESM6_ESM.pdf]

nature research

Corresponding author(s): Toshiyuki Okano

Last updated by author(s): May 3, 2019

Reporting Summary

Nature Research wishes to improve the reproducibility of the work that we publish. This form provides structure for consistency and transparency in reporting. For further information on Nature Research policies, see Authors & Referees and the Editorial Policy Checklist.

Statistics

For all statistical analyses, confirm that the following items are present in the figure legend, table legend, main text, or Methods section.

n/a	Confirmed	
☐	☒	The exact sample size (*n*) for each experimental group/condition, given as a discrete number and unit of measurement
☒	☐	A statement on whether measurements were taken from distinct samples or whether the same sample was measured repeatedly
☒	☐	The statistical test(s) used AND whether they are one- or two-sided<br>*Only common tests should be described solely by name; describe more complex techniques in the Methods section.*
☒	☐	A description of all covariates tested
☒	☐	A description of any assumptions or corrections, such as tests of normality and adjustment for multiple comparisons
☒	☐	A full description of the statistical parameters including central tendency (e.g. means) or other basic estimates (e.g. regression coefficient) AND variation (e.g. standard deviation) or associated estimates of uncertainty (e.g. confidence intervals)
☒	☐	For null hypothesis testing, the test statistic (e.g. *F*, *t*, *r*) with confidence intervals, effect sizes, degrees of freedom and *P* value noted<br>*Give P values as exact values whenever suitable.*
☒	☐	For Bayesian analysis, information on the choice of priors and Markov chain Monte Carlo settings
☒	☐	For hierarchical and complex designs, identification of the appropriate level for tests and full reporting of outcomes
☒	☐	Estimates of effect sizes (e.g. Cohen's *d*, Pearson's *r*), indicating how they were calculated

Our web collection on statistics for biologists contains articles on many of the points above.

Software and code

Policy information about availability of computer code

Data collection	n/a
Data analysis	Affinity constants were determined by using solver in Excel (Microsoft).<br>EC50 of the immunoreactions were calculated by curve fitting using ImageJ (NIH).<br>All software used for structure prediction and docking simulation were available online: ABodyBuilder (http://opig.stats.ox.ac.uk/webapps/sabdab-sabpred/Modelling.php); Kotai Antibody Builder (http://kotaiab.org/); PIGSPro (https://cassandra.med.uniroma1.it/pigspro/); PEP-FOLD 3.5 (http://bioserv.rpbs.univ-paris-diderot.fr/services/PEP-FOLD3/); PEPstrMOD (http://osddlinux.osdd.net/raghava/pepstrmod/); ClusPro 2.0 (https://cluspro.bu.edu/login.php); CABS-dock(http://biocomp.chem.uw.edu.pl/CABSdock); GalaxyPepDock (http://galaxy.seoklab.org/cgi-bin/submit.cgi?type=PEPDOCK); pepATTRACT (http://bioserv.rpbs.univ-paris-diderot.fr/services/pepATTRACT).<br>The molecular structures were rendered by using PyMOL 2.1.0 (Schrödinger).<br>The interaction sites in the docked complex were analyzed by LigPlot+.<br>Molecular dynamics simulations were performed by using NAMD 2.12 Win 64-CUDA and analyzed with VMD 1.9.3.

For manuscripts utilizing custom algorithms or software that are central to the research but not yet described in published literature, software must be made available to editors/reviewers. We strongly encourage code deposition in a community repository (e.g. GitHub). See the Nature Research guidelines for submitting code & software for further information.

Data

Policy information about availability of data

All manuscripts must include a data availability statement. This statement should provide the following information, where applicable:

- Accession codes, unique identifiers, or web links for publicly available datasets
- A list of figures that have associated raw data
- A description of any restrictions on data availability

The data supporting the findings of this study are available within the paper and its Supplementary Information files.

Field-specific reporting

Please select the one below that is the best fit for your research. If you are not sure, read the appropriate sections before making your selection.

☒ Life sciences ☐ Behavioural & social sciences ☐ Ecological, evolutionary & environmental sciences

For a reference copy of the document with all sections, see nature.com/documents/nr-reporting-summary-flat.pdf

Life sciences study design

All studies must disclose on these points even when the disclosure is negative.

Sample size	Sample sizes were indicated in the figure legends.
Data exclusions	n/a
Replication	All attempts at replication were successful.
Randomization	n/a
Blinding	n/a

Reporting for specific materials, systems and methods

We require information from authors about some types of materials, experimental systems and methods used in many studies. Here, indicate whether each material, system or method listed is relevant to your study. If you are not sure if a list item applies to your research, read the appropriate section before selecting a response.

Materials & experimental systems

n/a	Involved in the study	
☐	☒	Antibodies
☒	☐	Eukaryotic cell lines
☒	☐	Palaeontology
☐	☒	Animals and other organisms
☒	☐	Human research participants
☒	☐	Clinical data

Methods

n/a	Involved in the study	
☒	☐	ChIP-seq
☒	☐	Flow cytometry
☒	☐	MRI-based neuroimaging

Antibodies

Antibodies used	The anti-cCRY4 mAbs termed C1 to C15 were screened in our laboratory.<br>Anti 6×Histidine monoclonal antibody (Wako, clone 9F2),GST antibody (Santa Cruz, clone B-14), anti-mouse IgG (H+L) antibody, human serum adsorbed and peroxidase labeled (SeraCare Life Sciences, Inc., 074-1806), anti-mouse IgG, AP-linked antibody (Cell Signaling Technology, #7056S), IgG from mouse serum (Sigma, I5381-1MG) were commercial antibodies.
Validation	The validation statements of commercial antibodies were found on the manufacturer's websites.<br>Anti-cCRY4 antibodies were developed by used GST-fusion or MBP-fusion cCRY4CCE (Asp470–Thr529 of chicken CRY4) as the antigen. Epitopes of each monoclonal antibody were determined by competitive ELISA using synthetic peptides for cCRY4CCE. The dilution ratio when used in the experiment was determined by ELISA. In Western blotting, specificity of the antibodies was confirmed by control experiments using normal IgG instead of the antibodies.

Animals and other organisms

Policy information about studies involving animals; ARRIVE guidelines recommended for reporting animal research

Laboratory animals	Female BALB/c mice were used for immunization.
Wild animals	n/a
Field-collected samples	n/a
Ethics oversight	All studies were approved by the Committee for Animal Experimentation of the School of Science and Engineering at Waseda University (permission 2011-A073, 2012-A052, 2012-A055, WD11-84, WD12-079).

Note that full information on the approval of the study protocol must also be provided in the manuscript.
